# Biomass Productivity and Water Use Efficiency Are Elevated in Forage Crops Compared with Grain Crops in Hydrothermally Limited Areas

**DOI:** 10.3390/plants14243736

**Published:** 2025-12-08

**Authors:** Qiujin Ma, Fangyuan Yin, Xiaolong Zhou, Lin Wang, Kexuan Zhu, Xiaogang Li

**Affiliations:** 1 College of Ecology and Environment, Xinjiang University, Urumqi 830017, China; 107552405055@stu.xju.edu.cn (F.Y.); zhouxiaolong@xju.edu.cn (X.Z.); 20231209221@stu.xju.edu.cn (K.Z.); 2 Key Laboratory of Oasis Ecology, Ministry of Education, Xinjiang University, Urumqi 830017, China; 3 Ecological Postdoctoral Research Station, Xinjiang University, Urumqi 830017, China; 4 College of Ecology, Lanzhou University, Lanzhou 730000, China; wangl86@lzu.edu.cn (L.W.); lixiaogang@lzu.edu.cn (X.L.)

**Keywords:** forage crops, aboveground biomass, crude protein yield, water use efficiency, soil water storage

## Abstract

Insufficient precipitation and low temperatures can restrict grain yield but not necessarily vegetative growth in cold–arid regions. This indicates that forage production may be more suitable than grain cultivation in these environments while also meeting the increasing demand for livestock products. In this study, we compared the effects of cultivating forage maize (*Zea mays* L.) and forage oat (*Avena sativa* L.) with those of traditional grain crops, such as potato (*Solanum tuberosum* L.) and wheat (*Triticum aestivum* L.), in terms of aboveground biomass, crude protein yield, and water use efficiency (WUE). Across the four-year study, the results showed that aboveground biomass increased by 26–125% with oat (9.10 t ha^−1^) and maize (13.7 t ha^−1^) cultivation compared to potato (7.23 t ha^−1^) or wheat (6.10 t ha^−1^). Maize and potato exhibited greater biomass stability due to longer growing seasons and better synchronization with peak precipitation. In contrast, wheat and oat exhibited higher biomass variability, reflecting their susceptibility to early spring drought. Among the four crops analyzed, maize achieved the highest crude protein yield (1068 kg ha^−1^) and WUE (31.9 kg biomass ha^−1^ mm^−1^), primarily due to its superior biomass production rather than its protein concentration or elevated soil water consumption. Therefore, cultivating forage crops with longer growth periods could effectively align water demand with seasonal precipitation, thereby improving biomass accumulation and WUE in hydrothermally limited regions.

## 1. Introduction

Insufficient precipitation and low temperatures are critical factors limiting crop production in the cold and arid regions of the Loess Plateau [1,2]. Scarce precipitation and its irregular seasonal distribution typically result in crop drought [3]. Drought triggers a series of adverse physiological effects in crops. The structure and function of chlorophyll are compromised, stomatal conductance is diminished, and photosynthetic enzyme activity is reduced, thereby weakening photosynthesis and assimilate accumulation [4,5]. Exposure to drought at the critical stage of grain formation results in substantial decreases in crop yield [6,7,8,9]. For instance, drought during the flowering period of crops leads to reduced pollen vitality and pollination difficulties, resulting in fewer grains per ear [10,11]. Moreover, drought occurring prior to the grain-filling stage impedes the transport of photosynthetic products to the reproductive organs, resulting in an inadequate carbohydrate supply and a reduced grain-filling rate [12,13]. Additionally, for high-yielding crops, such as maize, low temperatures impede seedling emergence, extend the growing season, and may prevent grain filling before the first frost, ultimately reducing thousand-kernel weight and grain yield [14].

In the cold–arid regions of the Loess Plateau, cultivation is typically limited to low-yielding grain crops, including wheat (*Triticum aestivum* L.), potato (*Solanum tuberosum* L.), and pea (*Pisum sativum* L.), due to insufficient precipitation and low temperatures [15]. Over the past three decades, plastic film mulching technology has been widely adopted in agricultural production for its ability to mitigate soil hydrothermal limitations and enhance crop yields (mainly by improving the harvest index) [1,2,14]. However, using plastic films for mulching results in plastic debris accumulating in the soil, which can have adverse effects on crop growth and the soil environment [16,17,18]. Therefore, novel solutions should be investigated to maintain high productivity without using plastic film mulch, thereby supporting agricultural sustainability in this region.

In recent years, the rising demand for animal protein has prompted farmers to cultivate forage crops, such as maize (*Zea mays* L.) and oat (*Avena sativa* L.), for livestock feed [19,20]. This change in land use is better suited to the local climate, as sparse precipitation and low temperatures limit grain yield but do not necessarily limit vegetative growth in this region. Traditional crops are highly sensitive to drought and low temperatures during the critical grain formation period, resulting in highly unstable production [2,7,21]. However, forage production primarily focuses on harvesting the entire aboveground biomass, which has two key advantages. First, it eliminates the need to wait for full grain maturity, thereby reducing sensitivity to low temperatures. Second, it mitigates the risk of total crop failure in the event of severe drought during the critical grain formation stages. Therefore, in the high-altitude regions of the Loess Plateau, the hydrothermal conditions necessary for vegetative growth differ from those required for reproductive growth. This suggests that forage crops may exhibit greater yield stability than that observed for grain crops. Nevertheless, the impact of planting forage crops rather than traditional grain crops on agricultural productivity in cold–arid regions is rarely reported.

Soil water availability is a critical limiting factor in crop production in arid rain-fed agricultural areas. Crops demonstrate marked differences in their water requirements and sensitivity at various growth stages [22,23]. This highlights the need for a comprehensive analysis of the relationship between crop growth processes and soil moisture dynamics [21,24]. Soil water content is determined by the balance between precipitation and crop evapotranspiration (ET) and serves as an indicator of crop water stress and field water consumption [25]. The Loess Plateau has an uneven seasonal distribution of precipitation, with over 60% of annual rainfall typically falling from July to September, which often leads to drought conditions in spring [24,26]. Therefore, aligning crop water demand with seasonal precipitation is essential for enhancing agricultural productivity and water use efficiency (WUE) [27,28].

This study aimed to determine (1) whether planting forage crops (including maize and oat) increases aboveground biomass, crude protein yield, and WUE compared with planting traditional grain crops (wheat and potato) in hydrothermally limited areas on the Loess Plateau; and (2) whether maize and oat cultivation increases soil water consumption. We hypothesized that cultivating maize would primarily increase biomass, crude protein yield, and WUE by reducing the mismatch between seasonal precipitation distribution and peak water uptake rather than by increasing soil water depletion. We anticipate that the findings of this study will advance our understanding of how forage and grain crops differ in terms of biomass productivity, crude protein yield, and WUE under hydrothermally limited conditions and will provide a theoretical basis for optimizing crop selection and management strategies to enhance sustainable agricultural production in cold–arid regions.

## 2. Results

### 2.1. Precipitation

**Figure 1:**
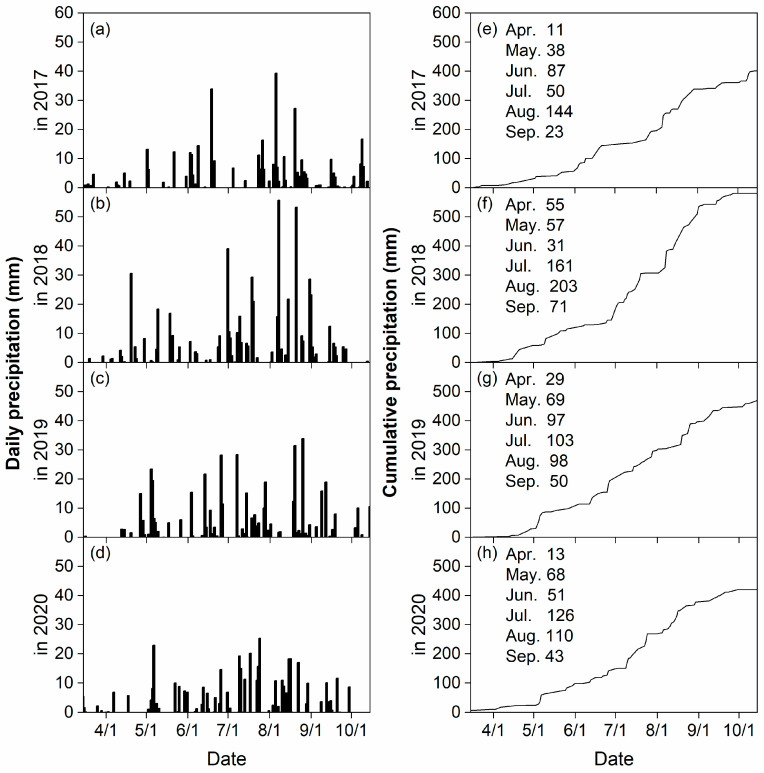
Daily and cumulative precipitation from 15 March to 15 October at the experimental site during the 2017 (**a**,**e**), 2018 (**b**,**f**), 2019 (**c**,**g**), and 2020 (**d**,**h**) growing seasons.

Cumulative precipitation from 15 March to 15 October was lowest in 2017 (401 mm), interim in 2019 (470 mm) and 2020 (420 mm), and highest in 2018 (581 mm) (Figure 1a–h). Across four years, cumulative precipitation in the growth period was 146–306 mm for wheat, 146–387 mm for oat, and 380–578 mm for both potato and maize.

### 2.2. Aboveground Biomass

**Figure 2:**
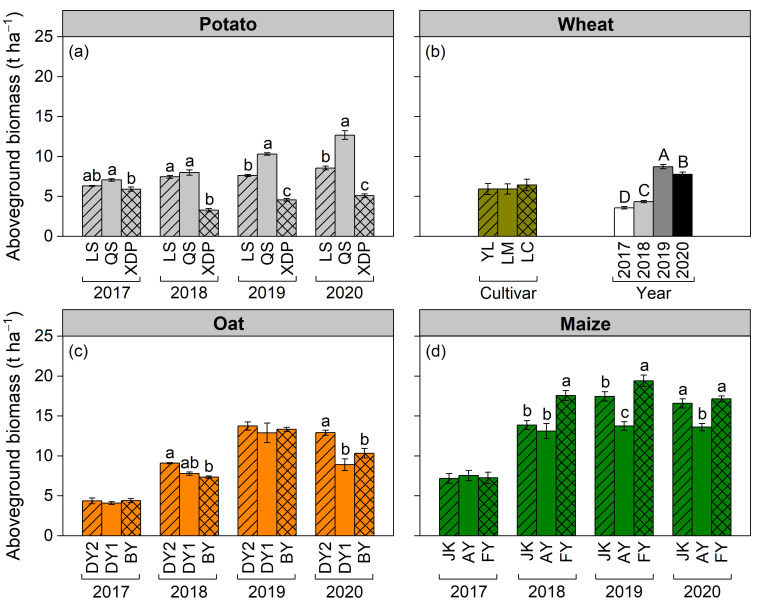
Effects of cultivar and cropping year on aboveground biomass (t ha^−1^) of potato (including tuber), wheat, oat, and maize. Panels (**a**,**c**,**d**) show cultivar effects on potato, oat, and maize biomass in the 2017, 2018, 2019, and 2020 cropping years (interactive effect), respectively; different lowercase letters indicate significant differences at *p* < 0.05 between cultivars in a year. Panel (**b**) shows cultivar and cropping year effects on wheat biomass (main effect); different uppercase letters indicate significant differences at *p* < 0.05 among cropping years. Bars represent ± one standard error (*n* = 3 for potato, oat, and maize cultivars, *n* = 12 for wheat cultivars, and *n* = 9 for cropping years). The potato cultivars of Longshu 7, Qingshu 9, and Xindaping are abbreviated as LS, QS, and XDP, respectively; wheat cultivars of Yongliang 15, Linmai 34, and Longchun 29 are abbreviated as YL, LM, and LC, respectively; oat cultivars of Dingyan 2, Dingyin 1, and Baiyan 7 are abbreviated as DY2, DY1, and BY, respectively; maize cultivars of Jinkai 5, Aoyu 5102, and Fangyu 36 are abbreviated as JK, AY, and FY, respectively.

The effects of cultivar on aboveground biomass varied across years in potato, oat, and maize, except for wheat (Figure 2a–d). In potato, the biomass of cultivars Longshu 7 (LS) and Qingshu 9 (QS) was higher than that of the cultivar Xindaping (XDP) in 2017 and 2018; however, the biomass ranking changed to QS > LS > XDP in 2019 and 2020 (Figure 2a). In oat, the biomass in cultivar Dingyan 2 (DY2) was larger than that in Dingyin 1 (DY1) and Baiyan 7 (BY) in 2018 and 2020; however, it was similar between the three cultivars in 2017 and 2019 (Figure 2c). In maize, the biomass did not differ between cultivars in 2017; however, it was higher in cultivar Fangyu 36 (FY) than that in cultivars Jinkai 5 (JK) and Aoyu 5102 (AY) in 2018, 2019, and 2020 (Figure 2d). Averaged over the four cropping years, the three cultivars of wheat had similar biomass (Figure 2b). Across cultivars and cropping years, aboveground biomass increased according to the following order: wheat (6.10 t ha^−1^) < potato (7.23 t ha^−1^, including tuber) < oat (9.10 t ha^−1^) < maize (13.7 t ha^−1^) (Figure 2a–d).

### 2.3. Crude Protein Yield and Its Variability Across Cropping Years

**Figure 3:**
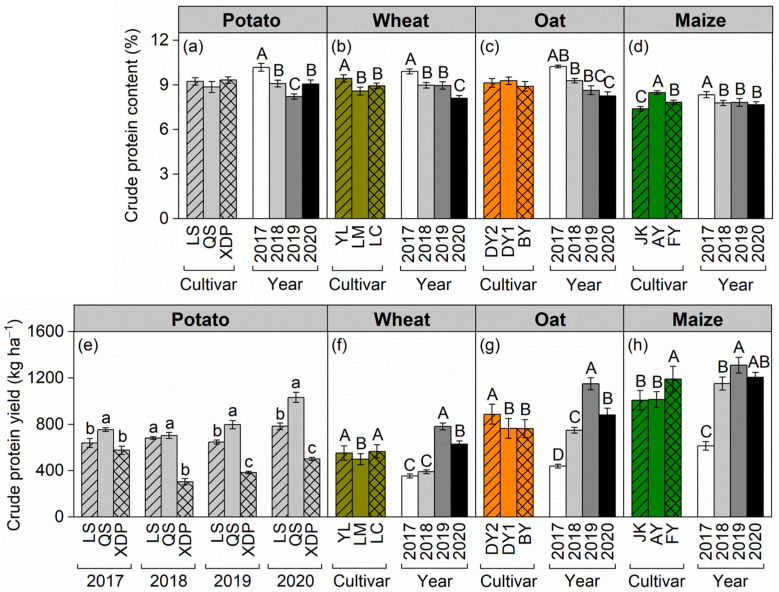
Effects of cultivar and cropping year on crude protein content (%) and crude protein yield (kg ha^−1^) of potato (including tuber) (**a**,**e**), wheat (**b**,**f**), oat (**c**,**g**), and maize (**d,h**). In panels (**a**–**d**,**f**–**h**), different uppercase letters indicate significant differences at *p* < 0.05 among crop cultivars or between cropping years (main effect) within each crop species; bars represent ± one standard error (*n* = 12 for potato, wheat, oat, and maize cultivars, *n* = 9 for cropping years). Panel (**e**) shows the effect of potato cultivar on crude protein yield in the 2017, 2018, 2019, and 2020 cropping years (interactive effect); different lowercase letters indicate significant differences at *p* < 0.05 between cultivars in a year. Bars represent ± one standard error (*n* = 3 for potato cultivars). The potato cultivars of Longshu 7, Qingshu 9, and Xindaping are abbreviated as LS, QS, and XDP, respectively; wheat cultivars of Yongliang 15, Linmai 34, and Longchun 29 are abbreviated as YL, LM, and LC, respectively; oat cultivars of Dingyan 2, Dingyin 1, and Baiyan 7 are abbreviated as DY2, DY1, and BY, respectively; maize cultivars of Jinkai 5, Aoyu 5102, and Fangyu 36 are abbreviated as JK, AY, and FY, respectively.

Cultivar and cropping year independently affected the crude protein content of aboveground biomass within each crop species (Figure 3a–d). Potato and oat exhibited similar crude protein contents in their biomass between cultivars (Figure 3a,c). Averaged over cropping years, in wheat, the biomass crude protein content was higher in cultivar Yongliang 15 (YL) than that in cultivars Linmai 34 (LM) and Longchun 29 (LC) (Figure 3b). In maize, the biomass crude protein content increased in the following order: JK < FY < AY (Figure 3d). Across all crop species, the biomass crude protein content was highest in 2017 among the four cropping years (Figure 3a–d). Across cultivars, the crude protein content of potato (9.13%), wheat (9.09%), and oat (9.10%) was generally higher than that of maize (7.90%) (Figure 3a–d).

The effect of cultivar on biomass crude protein yield varied by year exclusively in potato (Figure 3e–h). In potato, the cultivar QS exhibited a higher crude protein yield than that in cultivars LS and XDP in 2017, 2019, and 2020; however, the crude protein yield of cultivar LS was similar to that of cultivar QS, with both exceeding that of cultivar XDP in 2018 (Figure 3e). Cultivars YL and LC in wheat, DY2 in oat, and FY in maize performed better in crude protein yield (Figure 3f–h). Averaged across cultivars, the highest crude protein yield of wheat was observed in 2019, intermediate in 2020, and lowest in 2017 and 2018 (Figure 3f). The crude protein yield of oat and maize followed the order of 2019 > 2020 > 2018 > 2017 (Figure 3g,h). Across cultivars and cropping years, the highest crude protein yield was observed in maize (1068 kg ha^−1^), intermediate in oat (806 kg ha^−1^), and lowest in potato (650 kg ha^−1^) and wheat (538 kg ha^−1^) (Figure 3e–h).

**Figure 4:**
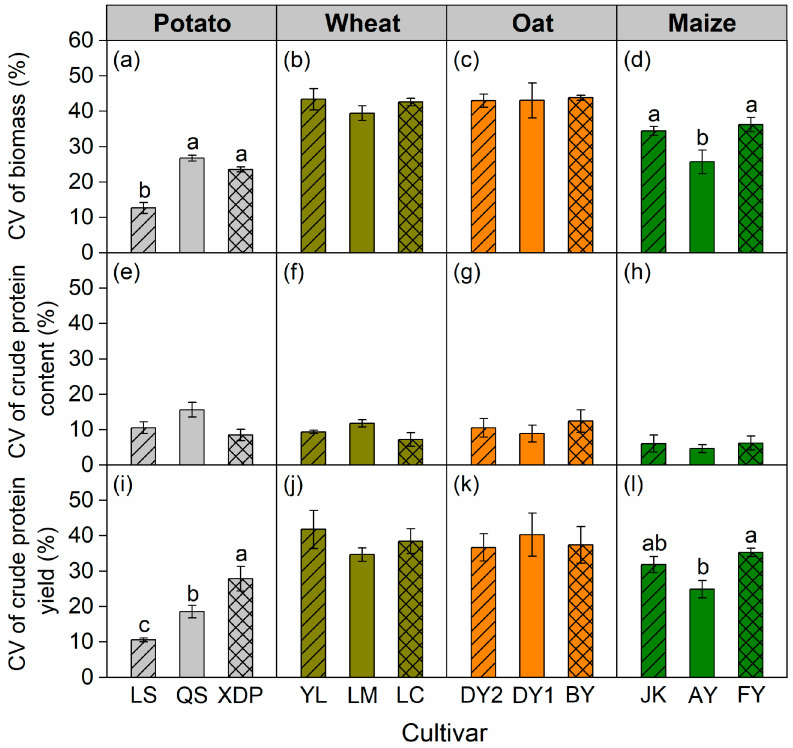
Coefficient of variation (*CV*) of biomass (**a**–**d**), crude protein content (**e**–**h**), and crude protein yield (**i**–**l**) over 2017–2020 cropping years for potato, wheat, oat, and maize cultivars. Bars represent ± one standard error (*n* = 12). Different lowercase letters indicate significant differences at *p* < 0.05 between cultivars within the same crop species. The potato cultivars of Longshu 7, Qingshu 9, and Xindaping are abbreviated as LS, QS, and XDP, respectively; wheat cultivars of Yongliang 15, Linmai 34, and Longchun 29 are abbreviated as YL, LM, and LC, respectively; oat cultivars of Dingyan 2, Dingyin 1, and Baiyan 7 are abbreviated as DY2, DY1, and BY, respectively; maize cultivars of Jinkai 5, Aoyu 5102, and Fangyu 36 are abbreviated as JK, AY, and FY, respectively.

The coefficient of variation (*CV*) for aboveground biomass and crude protein yield differed between cultivars in potato and maize over the years but remained consistent in wheat and oat (Figure 4a–d,i–l). Across cultivars, potato and maize had lower *CV* for biomass and crude protein yield than those in wheat or oat (Figure 4a–d,i–l). The *CV* of crude protein content is lower than that of biomass and crude protein yield (Figure 4a–l).

### 2.4. Soil Water Use by Crops and Water Use Efficiency

**Figure 5:**
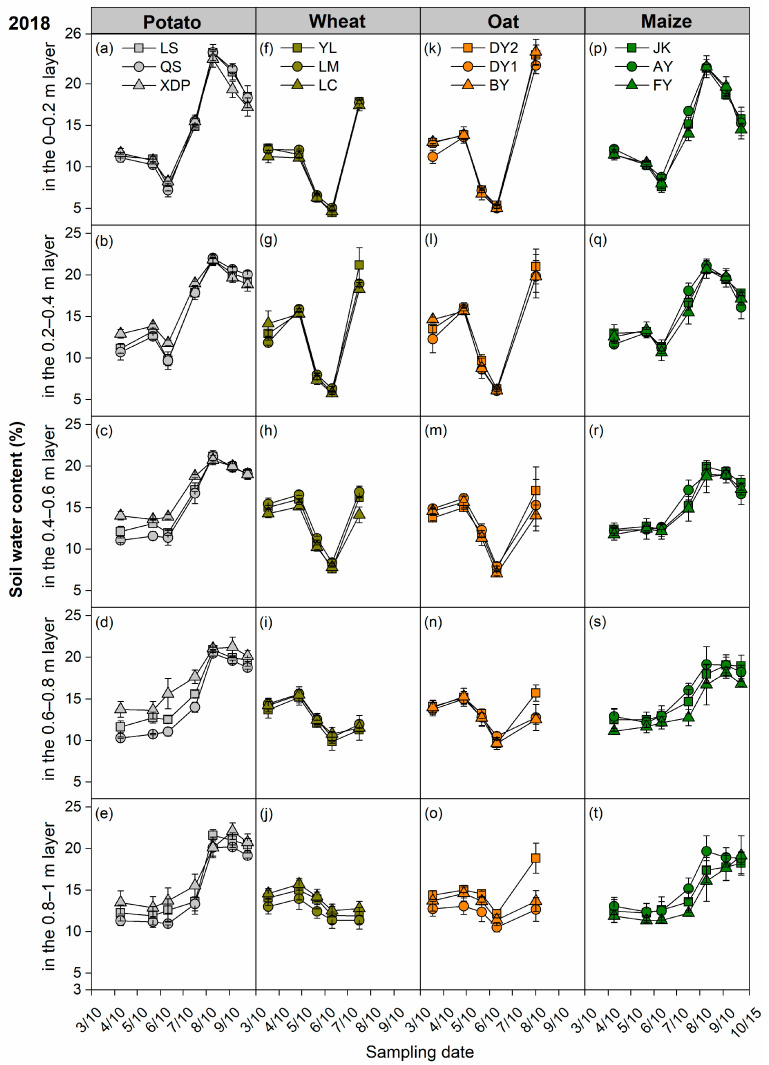
Soil water content dynamics in the 0–0.2 m, 0.2–0.4 m, 0.4–0.6 m, 0.6–0.8 m, 0.8–1 m layers for each cultivar of potato (**a**–**e**), wheat (**f**–**j**), oat (**k**–**o**), and maize (**p**–**t**) crops throughout the 2018 growing season. Bars represent ± one standard error (*n* = 3). The potato cultivars of Longshu 7, Qingshu 9, and Xindaping are abbreviated as LS, QS, and XDP, respectively; wheat cultivars of Yongliang 15, Linmai 34, and Longchun 29 are abbreviated as YL, LM, and LC, respectively; oat cultivars of Dingyan 2, Dingyin 1, and Baiyan 7 are abbreviated as DY2, DY1, and BY, respectively; maize cultivars of Jinkai 5, Aoyu 5102, and Fangyu 36 are abbreviated as JK, AY, and FY, respectively.

We monitored the soil water dynamics at 1 m depth for all crops in 2017, 2018, and 2019 (Figure S1, Figure 5 and Figure S2). The soil water content in the 0–0.2 m layer was close to or even lower than the permanent wilting point in both 2017 and 2018, but not in 2019 (Figure S1a,f,k,p, Figure 5a,f,k,p and Figure S2a,f,k,p). In 2017, this phenomenon was observed across all four crops, although the duration varied greatly. It lasted throughout the entire period from jointing to harvest for both wheat and oat, whereas it occurred during the tuber formation period in potato and before anthesis in maize (Figure S1a,f,k,p). In 2018, the phenomenon was observed exclusively in wheat (from jointing to filling stage) and oat (from jointing to before anthesis) (Figure 5f,k).

**Figure 6:**
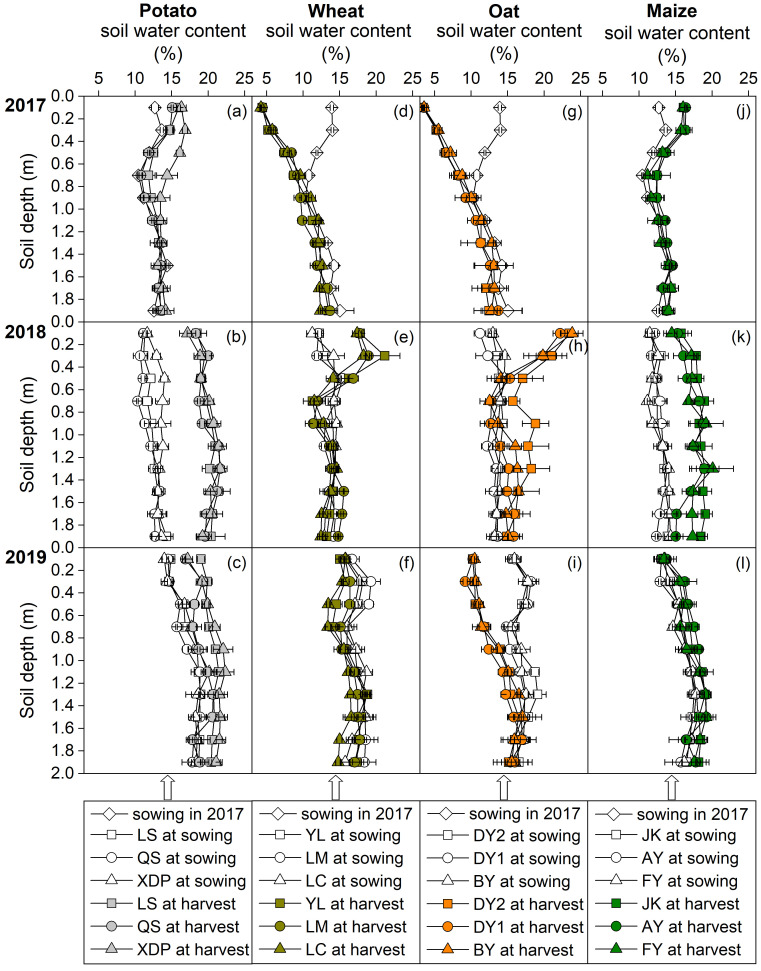
Soil water content in the 0–2 m profile at sowing and harvest for each cultivar of potato (**a**–**c**), wheat (**d**–**f**), oat (**g**–**i**), and maize (**j**–**l**) crops in 2017–2019 cropping years. Bars represent ± one standard error (*n* = 3). The potato cultivars of Longshu 7, Qingshu 9, and Xindaping are abbreviated as LS, QS, and XDP, respectively; wheat cultivars of Yongliang 15, Linmai 34, and Longchun 29 are abbreviated as YL, LM, and LC, respectively; oat cultivars of Dingyan 2, Dingyin 1, and Baiyan 7 are abbreviated as DY2, DY1, and BY, respectively; maize cultivars of Jinkai 5, Aoyu 5102, and Fangyu 36 are abbreviated as JK, AY, and FY, respectively.

The effects of cultivar and cropping year on soil water content and storage in the 0–2 m profile at sowing and harvest, total seasonal ET, and ET rate varied based on the crops (Figure 6a–l; Table 1 and Table S1). Across cultivars, the soil water storage (SWS) at sowing in 2019 was generally higher than that in 2017 and 2018 for all four crops (Table 1). In the same year, SWS was similar at sowing for all four crops; however, at harvest, maize and potato had higher SWS than wheat and oat (Table 1). Maize and potato exhibited higher ET throughout growing seasons than that for wheat and oat; however, ET rates were comparable among the four crops (Table 1).

**Figure 7:**
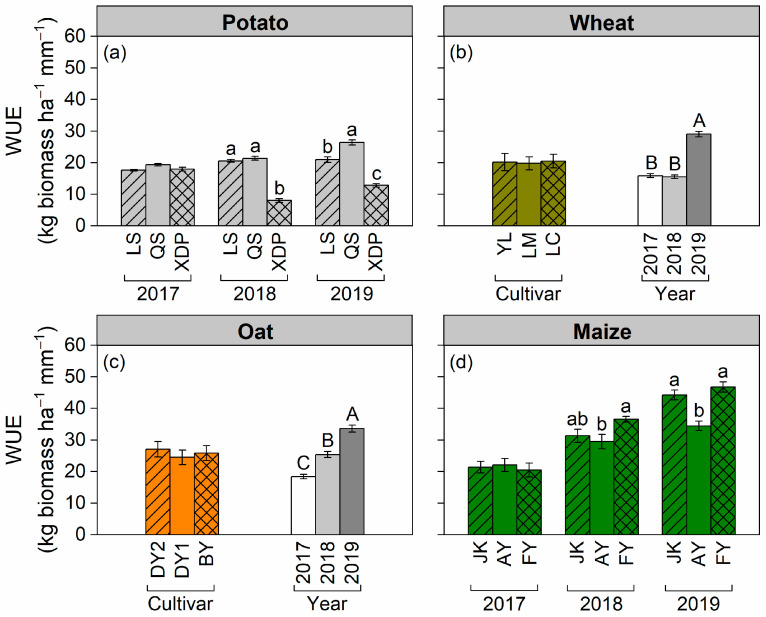
Effects of cultivar and cropping year on water use efficiency (WUE) of potato (including tuber), wheat, oat, and maize. Panels (**a**,**d**) show the effects of cultivar on potato and maize WUE in the 2017, 2018, and 2019 cropping years (interactive effect); different lowercase letters indicate significant differences at *p* < 0.05 between cultivars in a year within crop species. Panels (**b**,**c**) show the effects of cultivar and cropping year on wheat and oat WUE (main effect); different uppercase letters indicate significant differences at *p* < 0.05 between cropping years. Bars represent ± one standard error (*n* = 3 for potato and maize cultivars, *n* = 9 for wheat and oat cultivars and cropping years). The potato cultivars of Longshu 7, Qingshu 9, and Xindaping are abbreviated as LS, QS, and XDP, respectively; wheat cultivars of Yongliang 15, Linmai 34, and Longchun 29 are abbreviated as YL, LM, and LC, respectively; oat cultivars of Dingyan 2, Dingyin 1, and Baiyan 7 are abbreviated as DY2, DY1, and BY, respectively; maize cultivars of Jinkai 5, Aoyu 5102, and Fangyu 36 are abbreviated as JK, AY, and FY, respectively.

In potato and maize, the effects of cultivar on WUE changed with cropping year. Potato cultivar QS and maize FY exhibited higher WUE among different cultivars of the same crop in 2018 and 2019; however, WUE was similar between potato cultivars or maize cultivars in 2017 (Figure 7a,d). The WUE of both wheat and oat was influenced by the cropping year (higher in 2019 than in 2017 and 2018) and independent of the cultivar (Figure 7b,c). Across cultivars and cropping years, the WUE generally decreased along the sequence of maize (31.9 kg biomass ha^−1^ mm^−1^) > oat (25.8 kg biomass ha^−1^ mm^−1^) > wheat (20.1 kg biomass ha^−1^ mm^−1^) and potato (14.4 kg biomass ha^−1^ mm^−1^). Cultivating forage maize and oat significantly increased WUE compared with that of cultivating potato and wheat in rain-fed agricultural areas.

## 3. Discussion

### 3.1. Biomass Between Crop Species or Cultivars and Its Variability

This study demonstrates that forage crops (maize and oat) exhibit higher biomass in the cold–arid regions of the Loess Plateau compared with that observed for tuber potato and grain wheat. This suggests that in rain-fed agricultural areas facing hydrothermal limitations, cultivating maize and oat for forage is a viable strategy to improve agricultural productivity. While biomass production is widely recognized as being governed by the interaction of genetic potential and environmental conditions [2,20,29], our findings specifically highlight that the alignment between the peak crop water demand and seasonal distribution of precipitation, rather than the total precipitation amount, is the dominant factor controlling both the production and inter-annual stability of crop biomass. It is clearly illustrated by the contrasting crop responses to the highly uneven precipitation pattern of 2018. Despite receiving the highest rainfall during the 2018 growing season among the four years, the biomass of wheat and oat was significantly lower than that in 2019 and 2020 (Figure 2b,c). This was primarily due to the severe spring drought (April–June), which coincided with the critical growth stages of wheat and oat, thereby significantly constraining initial biomass accumulation. Although substantial precipitation occurred after anthesis, its effect on promoting biomass accumulation was limited. In contrast, maize biomass was less severely impacted by the spring drought in 2018 (Figure 2b–d), which is precisely because the peak water uptake stages of maize aligned with the period of abundant precipitation (July–August). The results underscore that phenological alignment with reliable rainfall periods is a critical determinant of the productivity of crops in regions with uneven seasonal precipitation, which is consistent with previous studies [19,21].

Beyond the critical role of phenological synchrony with precipitation, our results demonstrate the fundamental importance of the genetic characteristics of crops in determining biomass formation [30]. In this study, the superior biomass production of forage maize over the C_3_ crops is attributable to the inherent advantages of its C_4_ photosynthetic pathway, including higher photosynthetic capacity, greater resource-use efficiency, and enhanced drought tolerance [31,32,33,34]. Likewise, several other studies on crop biomass in northwest China have similarly reported a higher biomass potential in C_4_ crops [19,20]. Furthermore, even among C_3_ crops, genetic differences in canopy architecture drive significant variation in biomass. Oat consistently outperformed wheat, a difference attributable to its genetic propensity for greater plant height and enhanced tillering capacity. This architectural superiority allowed the oat to capture light more effectively and develop a larger photosynthetic area, ultimately supporting greater biomass production [35,36].

The results demonstrate that the *CV* of wheat and oat biomass exceeds potato and maize biomass (Figure 4a–d); this suggests that crops characterized by longer growth periods and later sowing dates exhibit greater biomass stability than those with shorter growth periods and earlier sowing dates. Short-growing-period crops, such as wheat (from April to July) and oat (from April to August), are particularly susceptible to drought from May to June; therefore, spring drought events typically result in a notable decrease in biomass [24,26]. The longer growing season of potato and maize enabled them to take advantage of the more reliable precipitation from July to September. The *CV* values of wheat, oat, and maize biomass were 41.8%, 43.3%, and 32.1%, respectively. The *CV* was generally high, primarily because of the restricted growth of all crops in 2017, resulting in biomass levels substantially lower than those observed in 2018–2020.

The observed biomass variation among cultivars of the same crop under identical climate conditions underscores the critical role of intraspecific genetic diversity in adapting to environmental constraints [37,38]. Potato cultivar QS and maize cultivar FY had higher biomass within the same crop in most of the experimental years (Figure 2a,d). In oat cropping, biomass levels were similar among cultivars in 2017 (the driest year) and 2019 (with higher water availability); however, oat cultivar DY2 biomass was higher than DY1 and BY biomass in 2018 and 2020 (Figure 2c), suggesting that DY2 possesses enhanced phenotypic plasticity adapted to erratic precipitation patterns. On the contrary, all wheat cultivars exhibited substantial inter-annual variability (Figure 2b), reflecting their general susceptibility to precipitation fluctuations.

### 3.2. Crude Protein Yielding

Crude protein content serves as a key indicator for assessing the nutritional value of forage crops [39,40,41]. The high crude protein content is essential for enhancing livestock growth and development, reducing feed costs, and improving the production efficiency of animal husbandry [42]. The nutrient acquisition ability of various crops or cultivars, akin to biomass formation, is influenced by their genetic factors and prevailing climate conditions [43]. This study demonstrated that the crude protein content across all cultivars of the same crop peaked in 2017, the driest year among four cropping years (Figure 3a–d). This may be attributed to the rapid decline in photosynthesis (biomass formation) under drought stress, outpacing the decline in nitrogen absorption. Our findings are consistent with those of Smit et al., who documented a significant increase in crude protein concentration in grasses under drought stress [44].

Crude protein yield is primarily influenced by biomass rather than protein concentration, both among crop species and across cropping years (Figure 3). Despite exhibiting the lowest crude protein content among the four crops, maize demonstrated the highest protein yield, primarily because of its largest biomass. In 2017, the driest year recorded, protein content was highest across all crops and varieties; however, crude protein yield was also the lowest due to constrained biomass accumulation. Therefore, these findings underscore that agronomic strategies aimed at maximizing and stabilizing biomass should be prioritized over those targeting protein concentration to ensure high and stable crude protein yield in forage production.

### 3.3. Water Use Efficiency

The scarcity of precipitation, substantial seasonal variability, and intense evaporation rate constitute the core challenges for semi-arid rain-fed agricultural areas; therefore, water emerges as the foremost limiting factor for agricultural production [45,46,47]. In this study, the frequency of soil water content in the 0–0.2 m layer approaching the permanent wilting point for wheat and oat was substantially higher than that for maize and potato in 2017 and 2018 (Figure 5 and Figure S1). This indicates that the mismatch between seasonal precipitation and crop water demand was more pronounced in wheat and oat than that in maize and potato. In addition, the total water ET of long-growth-period crops (maize and potato) exceeded that of short-growth-period crops (wheat and oat); however, the ET rates and SWS at the time of planting in the subsequent season were similar across the different crops (Table 1). This indicates that crops with longer growth periods did not increase soil water consumption. Moreover, the SWS of potato and maize is typically higher at harvest than that at sowing (Figure 6, Table 1). This implies that the precipitation input received during the growth period fully met the ET requirements of potato and maize, resulting in a surplus. This further demonstrates the strong alignment between the crop growth period and seasonal precipitation distribution for potato and maize.

The difference in WUE among crops and across cropping years was closely associated with changes in biomass productivity. Maize achieved the highest WUE among the four crops, primarily due to two factors: first, the C_4_ photosynthetic pathway reduces stomatal opening compared with the C_3_ photosynthetic pathway, thereby minimizing water evaporation loss [48]; second, the water requirement periods of maize are well synchronized with local seasonal precipitation, optimizing biomass production from late-season rainfall without increasing soil water consumption. WUE variations in crops across different growing seasons have been documented by numerous scholars [21,49,50]. Similarly to their research findings, the WUE of all crops and cultivars in this study showed significant inter-annual variations and generally followed the sequence of 2019 > 2018 > 2017 (Figure 7). Favorable soil water conditions at sowing and uniform precipitation during the growing season facilitated greater biomass accumulation and higher WUE in 2019.

In this study, we discussed how the alignment between crop water demand periods and seasonal precipitation patterns influences biomass production and WUE. A limitation of our approach is that it relied solely on measurements of total seasonal evapotranspiration and final biomass yield. To advance this understanding, future work should examine the dynamics of biomass accumulation, water consumption, and precipitation utilization at specific critical growth stages. Additionally, the generalizability of our findings is limited by the single-site conditions, the specific precipitation years encountered, and the restricted number of crop species tested. Therefore, subsequent research should employ multi-location trials, encompass a wider range of climatic conditions, and include a greater diversity of crops to validate and broaden the applicability of these results.

## 4. Materials and Methods

### 4.1. Study Site, Experimental Design and Management

**Figure 8:**
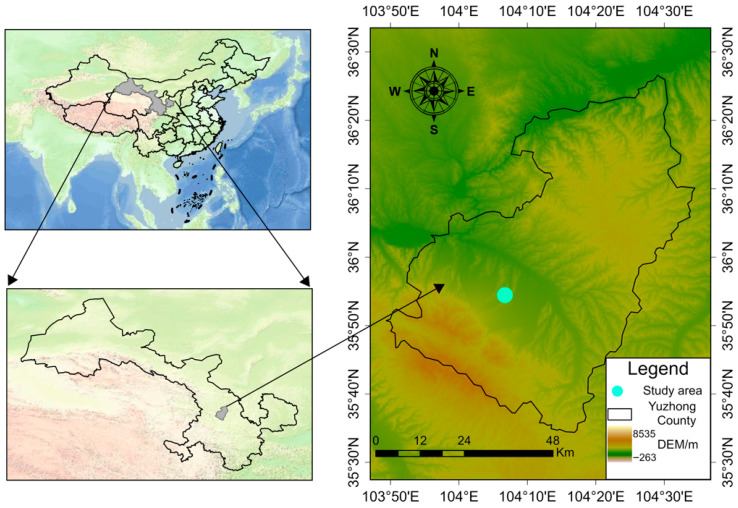
Location map of the study site.

The study was conducted in Yuzhong County (35°54′ N, 104°05′ E, *elev.* 2007 m), Gansu Province, northwest China, from 2017 to 2020 (Figure 8). The records of the nearest meteorological station indicate that the average annual precipitation at this experimental site over the past 30 years is 388 mm, with a mean annual air temperature of 6.7 °C. The soil formed from loess materials and is classified as Ustorthents in accordance with U.S. soil taxonomy. At the onset of the experiment in 2017, the soil was sampled pre-planting in the 0–0.2 m layer. The concentrations recorded were as follows: soil organic carbon concentration, 10.7 g kg^−1^; total nitrogen, 0.95 g kg^−1^; mineral nitrogen, 16.9 mg kg^−1^; total phosphorus, 0.90 g kg^−1^; and bulk density, 1.26 g cm^−3^. The permanent wilting point for the crops corresponded to a soil water content threshold of approximately 6.2% in the 0–0.2 m layer [51].

This experiment was designed to compare the biomass, crude protein yield, and WUE of forage crops, including maize and oat cultivars, with those of traditional grain crops, such as wheat and potato cultivars. A detailed description of the experimental design had been provided by Ma et al. [16]. Four main plots (each measuring 27.4 m long × 4.5 m wide) were randomly arranged to cultivate four crops (potato, wheat, oat, and maize). Subsequently, each main plot was subdivided into three subplots (each measuring 8.8 m long × 4.5 m wide) for the random planting of three crop cultivars, respectively. The isolation belts separating main plots or subplots measured 0.5 m in width. Treatments (a total of 12 cultivars from four crops) were replicated three times. In total, there were 36 subplots (4 crops × 3 cultivars × 3 replicates) in the field.

Three cultivars of each crop species were widely cultivated in the cold–arid regions of the Loess Plateau. The evaluated potato cultivars included Longshu 7 (abbreviated as LS, released in 2008), Qingshu 9 (QS, 2006), and Xindaping (XDP, 2005); the wheat cultivars consisted of Yongliang 15 (YL, 2000), Linmai 34 (LM, 2010), and Longchun 29 (LC, 2012); the oat cultivars comprised Dingyan 2 (DY2, 2016), Dingyin 1 (DY1, 2005), and Baiyan 7 (BY, introduced from Canada in the late 1990s); and the maize cultivars encompassed Jinkai 5 (JK, 2011), Aoyu 5102 (AY, 2004), and Fangyu 36 (FY, 2009).

The nitrogen fertilizer rate, phosphorus fertilizer rate, plant density, and sowing dates for each of the four crop species (potato, wheat, oat, and maize) during the 2017–2020 cropping years are presented in Table 2. The fertilizer application rates were based on the nutrient absorption characteristics of crops and prevailing local fertilization practices. Prior to winter, all subplots for the four crops were plowed to a depth of 0.2 m with a rotary tiller. During the experiment, no pesticides were used to control weeds, insects, and diseases.

### 4.2. Assessment of Biomass and Crude Protein Content

Annually, wheat and potato were harvested at the maturity stage (for grain or tuber, respectively), whereas maize and oat were harvested at the milky ripe stage (for silage). The harvest dates for four crop species across the 2017–2020 cropping years are presented in Table 2. The biomass sampling methods differed among crop species. For wheat and oat crops, two adjacent crop rows were harvested at ground level from three random locations within each subplot. For maize, all plants in two randomly selected adjacent rows within each subplot were cut off at ground level. The same method was employed to collect the stems and leaves of potato, and then the corresponding tubers were excavated; consequently, potato biomass in this study included stems, leaves, and tubers. All plant samples were oven-dried at 60 °C to constant weight and subsequently weighed.

After the various plant samples were ground into powder, they were digested in concentrated H_2_SO_4_ and H_2_O_2_ [52]. The total nitrogen in the digest was distilled using the Kjeldahl method [53]. Crude protein content was calculated by multiplying the nitrogen concentration by 6.25. Crude protein yield for each cultivar of the four crops was calculated as follows:

(1) Crude protein yield (kg ha^−1^) = Biomass (t ha^−1^) × Crude protein content (%) × 10

We calculated the *CV* for aboveground biomass, crude protein content, and crude protein yield in every subplot to assess the variation (or stability) of these traits across different cultivars of each crop species over the cropping years based on the following formula [54]:

(2) *CV* (%) = (*S*/*M*) × 100

where *M* is the overall mean of all observations for each cultivar across cropping years and replications; and *S* is the corresponding standard deviation.

### 4.3. Measurements of Soil Water Storage (SWS) and Crop Water Use Efficiency (WUE)

Soil water contents were measured at 0.2 m intervals to 2 m depth during sowing and harvest of each crop in 2017, 2018, and 2019, as well as at 0.2 m intervals to 1 m depth during the growth stage. Soils were randomly sampled from each subplot at three points using an auger (40 mm inner diameter). Soils were sampled within the rows of the wheat or oat subplots after the crops were cut from the land surface. Soils were sampled close to the stems of plants in the potato or maize subplots. Subsamples at the same depth were pooled to form one composite sample for each subplot. The soil gravimetric water content of a soil layer was determined by weighing the sample as taken from the field and after drying to constant weight at 105 °C. Annually, SWS (mm) in the 0–2 m profile was calculated from soil gravimetrical water content ( w, %) using the following equation:

(3) $$SWS=\sum_{i=1}^{n}w_{i}\times \rho_{i}\times h_{i}$$

where $\rho_{i}$ is the bulk density of soil layer *i*; $h_{i}$ is the depth of soil layer *i*; and *n* is the total number of soil layers.

Total seasonal rainfall was determined by summing measurements from individual precipitation events, recorded with a rain gauge (SDM6, Tianjin Meteorological Instrument Factory, Tianjin, China). The flat terrain and generally low precipitation intensity of the experimental field resulted in negligible runoff during the experimental period [19]. Therefore, crop water consumption during the entire growing season (evapotranspiration, ET) was calculated using the following equation:

(4) ET=P−∆SWS

where P is the cumulative precipitation (mm); and ∆SWS is the variation in *SWS* within the 2 m profile from sowing to harvest (mm).

The WUE (kg biomass ha^−1^ mm^−1^) for each cultivar of the four crops was calculated as follows:

(5) WUE=Biomass/ET×1000

where Biomass is the dry aboveground matter yield (t ha^−1^); and ET is the cumulative water loss via evapotranspiration over the whole growing season (mm).

### 4.4. Statistical Analysis

Two-way analysis of variance (ANOVA), utilizing cultivar and cropping year as two fixed factors, was used to assess the variation in biomass, crude protein content and yield, SWS, ET and ET rate, and WUE within a crop species. One-way ANOVA was employed to assess the effects of cultivar on the *CV* of biomass, *CV* of crude protein content, and *CV* of crude protein yield within each crop species. The significance of the differences between the means was determined using the least significant difference (LSD) test at *p* ≤ 0.05. All statistical procedures were performed using SPSS^®^ statistics software 27.0 (IBM Corporation, Armonk, NY, USA).

## 5. Conclusions

The cultivation of forage crops (maize and oat) increased aboveground biomass, crude protein yield, and WUE when compared with those of grain wheat and tuber potato. Crops with short growth periods, such as wheat and oat, were more susceptible to drought during early spring. Conversely, crops with longer growth periods and later sowing dates exhibited greater biomass stability, primarily due to the alignment of their peak water uptake stages with the local precipitation distribution. Our study demonstrates that cultivation of forage crops can enhance biomass productivity and optimize resource-utilization efficiency in the cold–arid regions of the Loess Plateau, which is of great significance for maintaining sustainable agricultural development.

## Figures and Tables

**Table 1:** Soil water storage (SWS) in the 0–2 m profile at sowing and harvest, change in SWS between sowing and harvest (ΔSWS), total seasonal evapotranspiration (ET), and ET rate as affected by potato, wheat, oat, and maize cultivars in 2017–2019 cropping years. The potato cultivars of Longshu 7, Qingshu 9, and Xindaping are abbreviated as LS, QS, and XDP, respectively; wheat cultivars of Yongliang 15, Linmai 34, and Longchun 29 are abbreviated as YL, LM, and LC, respectively; oat cultivars of Dingyan 2, Dingyin 1, and Baiyan 7 are abbreviated as DY2, DY1, and BY, respectively; maize cultivars of Jinkai 5, Aoyu 5102, and Fangyu 36 are abbreviated as JK, AY, and FY, respectively.

Crop	Cultivar	SWS at Sowing (mm)			SWS at Harvest (mm)			∆SWS (mm)			ET (mm)			ET Rate (mm d^−1^)		
		2017	2018	2019	2017	2018	2019	2017	2018	2019	2017	2018	2019	2017	2018	2019
Potato	LS	321	312	445	341	532	518	20.5	220	73.3	359	358	365	2.11	2.17	2.29
	QS	321	301	433	335	506	480	14.2	205	47.0	365	373	391	2.15	2.26	2.46
	XDP	321	338	445	371	506	527	49.9	168	81.4	330	410	357	1.94	2.49	2.24
Wheat	YL	328	353	440	248	377	415	−80.1	24.0	−24.7	226	282	291	2.13	2.39	2.34
	LM	328	352	454	247	384	421	−80.6	31.7	−29.6	227	274	296	2.14	2.32	2.38
	LC	328	349	437	252	371	387	−76.2	21.5	−49.5	222	284	316	2.10	2.41	2.54
Oat	DY2	328	349	436	236	412	342	−92.0	62.6	−93.7	238	324	398	2.12	2.35	2.90
	DY1	328	334	441	237	404	335	−90.9	70.3	−106	237	317	410	2.11	2.29	2.99
	BY	328	351	427	248	422	347	−82.8	70.6	−80.0	229	316	384	2.04	2.29	2.80
Maize	JK	321	331	409	365	464	452	44.0	133	43.5	335	445	394	1.97	2.69	2.48
	AY	321	325	402	358	458	441	36.8	133	39.0	343	445	399	2.02	2.70	2.51
	FY	321	326	407	345	424	430	24.6	97.8	23.2	355	480	415	2.09	2.91	2.61

**Table 2:** Details of sowing pattern, the rate of Nitrogen (N) and phosphorus (P) fertilizer, plant density, row and plant spacing, dates of sowing and harvest for four crop species (potato, wheat, oat, maize) in 2017–2020 cropping years.

Crop Species	Potato	Wheat	Oat	Maize
Sowing pattern	Hill-drop	In drill	In drill	Hill-drop
Urea (kg N ha^−1^)	140	105	105	210
Superphosphate (kg P ha^−1^)	23.6	15.7	15.7	31.4
Plant density (plants ha^−1^)	6.06 × 10^4^	450 × 10^4^	450 × 10^4^	7.58 × 10^4^
Row spacing (cm)	55	15	15	55
Plant spacing (cm)	30	-	-	24
Sowing date				
in 2017	25 April	2 April	2 April	25 April
in 2018	18 April	26 March	26 March	18 April
in 2019	22 April	24 March	24 March	22 April
in 2020	25 April	9 April	9 April	25 April
Harvest date				
in 2017	12 October	16 July	22 July	12 October
in 2018	30 September	22 July	11 August	30 September
in 2019	28 September	26 July	8 August	28 September
in 2020	7 October	31 July	8 August	7 October

## Data Availability

The original contributions presented in this study are included in the article and supplementary material. Further inquiries can be directed to the corresponding author.

## Supplementary Materials

The following supporting information can be downloaded at: https://www.mdpi.com/article/10.3390/plants14243736/s1. Figure S1: Soil water content dynamics in the 0–0.2 m, 0.2–0.4 m, 0.4–0.6 m, 0.6–0.8 m, 0.8–1 m layers for each cultivar of potato (a–e), wheat (f–j), oat (k–o), and maize (p–t) crops throughout the 2017 growing season. Bars represent ± one standard error (*n* = 3); Figure S2: Soil water content dynamics in the 0–0.2 m, 0.2–0.4 m, 0.4–0.6 m, 0.6–0.8 m, 0.8–1 m layers for each cultivar of potato (a–e), wheat (f–j), oat (k–o), and maize (p–t) crops throughout the 2019 growing season. Bars represent ± one standard error (*n* = 3); Table S1: Two-way ANOVA analysis of soil water storage (SWS) in the 0–2 m profile at sowing and harvest, change in SWS between sowing and harvest (ΔSWS), total seasonal evapotranspiration (ET) and ET rate under cultivation of potato, wheat, oat and maize in 2017–2019 cropping years.

## Abbreviations

The following abbreviations are used in this manuscript:

AY	Aoyu 5102 (maize cultivar)
BY	Baiyan 7 (oat cultivar)
CV	Coefficient of variation
DY1	Dingyin 1 (oat cultivar)
DY2	Dingyan 2 (oat cultivar)
ET	Evapotranspiration
FY	Fangyu 36 (maize cultivar)
JK	Jinkai 5 (maize cultivar)
LC	Longchun 29 (wheat cultivar)
LM	Linmai 34 (wheat cultivar)
LS	Longshu 7 (potato cultivar)
QS	Qingshu 9 (potato cultivar)
SWS	Soil water storage
WUE	Water use efficiency
XDP	Xindaping (potato cultivar)
YL	Yongliang 15 (wheat cultivar)

## References

[1] Wang N., Chu X., Li J., Luo X., Ding D., Siddique K.H.M., Feng H. (2025). Understanding Increased Grain Yield and Water Use Efficiency by Plastic Mulch from Water Input to Harvest Index for Dryland Maize in China’s Loess Plateau. Eur. J. Agron..

[2] Wang Y.P., Li X.G., Zhu J., Fan C.Y., Kong X.J., Turner N.C., Siddique K.H.M., Li F.-M. (2016). Multi-Site Assessment of the Effects of Plastic-Film Mulch on Dryland Maize Productivity in Semiarid Areas in China. Agric. For. Meteorol..

[3] Uslu Ö.S., Gedik O., Kaya A.R., Erol A., Babur E., Khan H., Seleiman M.F., Wasonga D.O. (2025). Effects of Different Irrigation Water Sources Contaminated with Heavy Metals on Seed Germination and Seedling Growth of Different Field Crops. Water.

[4] Flexas J., Diaz-Espejo A., Galmés J., Kaldenhoff R., Medrano H., Ribas-Carbo M. (2007). Rapid Variations of Mesophyll Conductance in Response to Changes in CO_2_ Concentration around Leaves. Plant Cell Environ..

[5] Hussain S., Hussain S., Qadir T., Khaliq A., Ashraf U., Parveen A., Saqib M., Rafiq M. (2019). Drought stress in plants: An Overview on Implications, Tolerance Mechanisms and Agronomic Mitigation Strategies. Plant Sci. Today.

[6] Adimassu Z., Mul M., Owusu A. (2023). Intra-Seasonal Rainfall Variability and Crop Yield in the Upper East Region of Ghana. Environ. Dev. Sustain..

[7] Madamombe S.M., Nyamadzawo G., Öborn I., Smucker A., Chirinda N., Kihara J., Nkurunziza L. (2025). Seasonal Rainfall Patterns Affect Rainfed Maize Production More than Management of Soil Moisture and Different Plant Densities on Sandy Soils of Semi-Arid Regions. Field Crops Res..

[8] Mbanyele V., Mtambanengwe F., Nezomba H., Groot J.C.J., Mapfumo P. (2021). Combinations of In-Field Moisture Conservation and Soil Fertility Management Reduce Effect of Intra-Seasonal Dry Spells on Maize under Semi-Arid Conditions. Field Crops Res..

[9] Diatta A.A., Abaye O., Battaglia M.L., Leme J.F.D.C., Seleiman M., Babur E., Thomason W.E. (2024). Mungbean [*Vigna radiata* (L.) Wilczek] and Its Potential for Crop Diversification and Sustainable Food Production in Sub-Saharan Africa: A review. Technol. Agron..

[10] Li H., Tang Y., Meng F., Zhou W., Liang W., Yang J., Wang Y., Wang H., Guo J., Yang Q. (2025). Transcriptome and Metabolite Reveal the Inhibition Induced by Combined Heat and Drought Stress on the Viability of Silk and Pollen in Summer Maize. Ind. Crops Prod..

[11] Shen S., Liang X., Zhang L., Zhao X., Liu Y., Lin S., Gao Z., Wang P., Wang Z., Zhou S. (2020). Intervening in Sibling Competition for Assimilates by Controlled Pollination Prevents Seed Abortion under Postpollination Drought in Maize. Plant Cell Environ..

[12] Shen S., Zhang L., Liang X.-G., Zhao X., Lin S., Qu L.-H., Liu Y.-P., Gao Z., Ruan Y.-L., Zhou S.-L. (2018). Delayed Pollination and Low Availability of Assimilates Are Major Factors Causing Maize Kernel Abortion. J. Exp. Bot..

[13] Tang Y., Guo J., Jagadish S.V.K., Yang S., Qiao J., Wang Y., Xie K., Wang H., Yang Q., Deng L. (2023). Ovary Abortion in Field-Grown Maize under Water-Deficit Conditions Is Determined by Photo-Assimilation Supply. Field Crops Res..

[14] Wang L., Li X.G., Guan Z.-H., Jia B., Turner N.C., Li F.-M. (2018). The Effects of Plastic-Film Mulch on the Grain Yield and Root Biomass of Maize Vary with Cultivar in a Cold Semiarid Environment. Field Crops Res..

[15] Ma Q., Luan F., Jia B., Zhang Q., Wang L., Cui Z., Li X.G. (2023). Agricultural Soil Aggregation Is Affected by the Crop Root Biomass Rather than Morphological Characteristics. J. Plant Nutr. Soil Sci..

[16] Gu X., Yin R., Cai W., Chen P., Cui K., Du Y., Li Y., Cai H. (2024). Residual Plastic Film Decreases Crop Yield and Water Use Efficiency through Direct Negative Effects on Soil Physicochemical Properties and Root Growth. Sci. Total Environ..

[17] Wang D., Xi Y., Shi X., Guo C., Zhong Y., Song C., Guan Y., Huang L., Yang Q., Li F. (2023). Effects of Residual Plastic Film on Crop Yield and Soil Fertility in a Dryland Farming System. J. Integr. Agric..

[18] Wang D., Xi Y., Shi X.-Y., Zhong Y.-J., Guo C.-L., Han Y.-N., Li F.-M. (2021). Effect of Plastic Film Mulching and Film Residues on Phthalate Esters Concentrations in Soil and Plants, and its Risk Assessment. Environ. Pollut..

[19] Zhang Q., Bell L.W., Shen Y., Whish J.P.M. (2018). Indices of Forage Nutritional Yield and Water Use Efficiency amongst Spring-Sown Annual Forage Crops in North-West China. Eur. J. Agron..

[20] Zhang Z., Whish J.P.M., Bell L.W., Nan Z. (2017). Forage Production, Quality and Water-Use-Efficiency of Four Warm-Season Annual Crops at Three Sowing Times in the Loess Plateau Region of China. Eur. J. Agron..

[21] Zhang Q., Wang S., Sun Y., Zhang Y., Li H., Liu P., Wang X., Wang R., Li J. (2022). Conservation Tillage Improves Soil Water Storage, Spring Maize (Zea Mays L.) Yield and WUE in Two Types of Seasonal Rainfall Distributions. Soil Tillage Res..

[22] Monteleone B., Borzí I., Bonaccorso B., Martina M. (2022). Developing Stage-Specific Drought Vulnerability Curves for Maize: The Case Study of the Po River basin. Agric. Water Manag..

[23] Zhu X., Xu K., Liu Y., Guo R., Chen L. (2021). Assessing the Vulnerability and Risk of Maize to Drought in China Based on the AquaCrop Model. Agric. Syst..

[24] Ren A., Zhao W., Sumera A., Lin W., Ding P., Hao R., Wang P., Zhong R., Tong J., Gao Z. (2022). Effects of Tillage and Seasonal Variation of Rainfall on Soil Water Content and Root Growth Distribution of Winter Wheat under Rainfed Conditions of the Loess Plateau, China. Agric. Water Manag..

[25] Vujić S., Krstić D., Mačkić K., Čabilovski R., Radanović Z., Zhan A., Ćupina B. (2021). Effect of Winter Cover Crops on Water Soil Storage, Total Forage Production, and Quality of Silage Corn. Eur. J. Agron..

[26] Sun M., Ren A., Gao Z., Wang P., Mo F., Xue L., Lei M. (2018). Long-Term Evaluation of Tillage Methods in Fallow Season for Soil Water Storage, Wheat Yield and Water Use Efficiency in Semiarid Southeast of the Loess Plateau. Field Crops Res..

[27] Feng Y., Guo Y., Shen Y., Zhang G., Wang Y., Chen X. (2024). Change of Crop Structure Intensified Water Supply-Demand Imbalance in China’s Black Soil Granary. Agric. Water Manag..

[28] Li R., Chai S., Chai Y., Li Y., Lan X., Ma J., Cheng H., Chang L. (2021). Mulching Optimizes Water Consumption Characteristics and Improves Crop Water Productivity on the Semi-Arid Loess Plateau of China. Agric. Water Manag..

[29] Hatfield J.L., Dold C. (2018). Agroclimatology and Wheat Production: Coping with Climate Change. Front. Plant Sci..

[30] Lin G.-Z., Chiang L.-C. (2025). Crop Conversion as a Strategy for Enhancing Water Efficiency and Nutrient Management under Climate Change. J. Hydrol..

[31] Mbava N., Mutema M., Zengeni R., Shimelis H., Chaplot V. (2020). Factors Affecting Crop Water Use Efficiency: A Worldwide Meta-Analysis. Agric. Water Manag..

[32] Cui H. (2021). Challenges and Approaches to Crop Improvement Through C3-to-C4 Engineering. Front. Plant Sci..

[33] Wang Y., Liu S., Shi H. (2023). Comparison of Climate Change Impacts on the Growth of C3 and C4 Crops in China. Ecol. Inform..

[34] Anapalli S.S., Fisher D.K., Reddy K.N., Krutz J.L., Pinnamaneni S.R., Sui R. (2019). Quantifying Water and CO2 Fluxes and Water Use Efficiencies across Irrigated C3 and C4 Crops in a Humid Climate. Sci. Total Environ..

[35] Martins T.S., Magalhães Filho J.R., Cruz L.P., Almeida R.L., Marchiori P.E.R., Silva A.L.B.O., Pires R.C.M., Landell M.G.A., Xavier M.A., Machado E.C. (2025). Light Interception and Conversion Efficiencies and Biomass Partitioning in Sugarcane Varieties with Varying Canopy Architecture under Subtropical Conditions. Field Crops Res..

[36] Li P., Yin W., Zhao L., Wan P., Fan Z., Hu F., Nan Y., Sun Y., Fan H., He W. (2025). No Tillage with Straw Mulching Enhanced Radiation Use Efficiency of Wheat via Optimizing Canopy Radiation Interception and Photosynthetic Properties. Field Crops Res..

[37] Zhuang H., Zhang Z., Xu J., Han J., Cheng F., Tao F. (2025). Overcoming Wheat Yield Stagnation in China Depends More on Cultivar Improvements than Water and Fertilizer Management. Field Crops Res..

[38] Hao B., Ma J., Si S., Wang X., Wang S., Li F., Jiang L. (2024). Response of Grain Yield and Water Productivity to Plant Density in Drought-Tolerant Maize Cultivar under Irrigated and Rainfed Conditions. Agric. Water Manag..

[39] Liu W., Yan S., Jiang N., Li M., Yin L., Zhang S., Xie X., Gao G., Chang S., Hou F. (2025). Interaction of Nitrogen and Mowing Frequency in Enhancing Regeneration and Crude Protein Content of Forage Oat in Northwestern of China. Field Crops Res..

[40] Zhang J., Hou S., Usman M., Hou F., Nan Z. (2025). [SI_Forage] Trade-Offs between Artificial Forage Yield and Quality under Multiple Mowing Enhance Grass-Livestock System Balance on the Qinghai-Tibetan Plateau. J. Integr. Agric..

[41] Larsen S.U., Manevski K., Lærke P.E., Jørgensen U. (2024). Biomass Yield, Crude Protein Yield and Nitrogen Use Efficiency over Nine Years in Annual and Perennial Cropping Systems. Eur. J. Agron..

[42] Uslu O.S., Babur E., Battaglia M.L., Turkkaya E., Seleiman M.F., Roy R., Dindaroglu T. (2023). Effects of Gyttja Applications on Hay Yield and Quality of a Rangeland in the Mediterranean Region. Int. J. Environ. Sci. Technol..

[43] Lammerts Van Bueren E.T., Thorup-Kristensen K., Leifert C., Cooper J.M., Becker H.C. (2014). Breeding for Nitrogen Efficiency: Concepts, Methods, and Case Studies. Euphytica.

[44] Smit M., Malan P., Smit N., Deacon F. (2025). Drought Impact on the Nutrients of Forage Plants in a Semi-Arid Rangeland and Its Potential Implications for Sustaining Herbivores. J. Arid Environ..

[45] Deng J., Zhang Z., Liang Z., Li Z., Yang X., Wang Z., Coulter J.A., Shen Y. (2020). Replacing Summer Fallow with Annual Forage Improves Crude Protein Productivity and Water Use Efficiency of the Summer Fallow-Winter Wheat Cropping System. Agric. Water Manag..

[46] Hou X., Li R. (2019). Interactive Effects of Autumn Tillage with Mulching on Soil Temperature, Productivity and Water Use Efficiency of Rainfed Potato in Loess Plateau of China. Agric. Water Manag..

[47] Zhang G., Mo F., Shah F., Meng W., Liao Y., Han J. (2021). Ridge-Furrow Configuration Significantly Improves Soil Water Availability, Crop Water Use Efficiency, and Grain Yield in Dryland Agroecosystems of the Loess Plateau. Agric. Water Manag..

[48] Way D.A., Katul G.G., Manzoni S., Vico G. (2014). Increasing Water Use Efficiency along the C3 to C4 Evolutionary Pathway: A Stomatal Optimization Perspective. J. Exp. Bot..

[49] Gheysari M., Loescher H.W., Sadeghi S.H., Mirlatifi S.M., Zareian M.J., Hoogenboom G., Sparks D.L. (2015). Chapter Three—Water-Yield Relations and Water Use Efficiency of Maize Under Nitrogen Fertigation for Semiarid Environments: Experiment and Synthesis. Advances in Agronomy.

[50] Wang S., Wang H., Zhang Y., Wang R., Zhang Y., Xu Z., Jia G., Wang X., Li J. (2018). The Influence of Rotational Tillage on Soil Water Storage, Water Use Efficiency and Maize Yield in Semi-Arid Areas under Varied Rainfall Conditions. Agric. Water Manag..

[51] Liu X.E., Li X.G., Hai L., Wang Y.P., Li F.M. (2014). How Efficient Is Film Fully-Mulched Ridge–Furrow Cropping to Conserve Rainfall in Soil at a Rainfed Site?. Field Crops Res..

[52] Lu R. (2000). Soil and Agro-Chemistry Analysis.

[53] Horwitz W. (1980). Official Methods of Analysis of the Association of Official Analytical Chemists.

[54] Quinn G.P., Keough M.J. (2002). Experimental Design and Data Analysis for Biologists.

