# Supplementary material for: Biomass Productivity and Water Use Efficiency Are Elevated in Forage Crops Compared with Grain Crops in Hydrothermally Limited Areas
Source: Plants (Basel). 2025 Dec 8;14(24):3736. doi: 10.3390/plants14243736 (PMC12737194; doi:10.3390/plants14243736)
Supplement: Supplementary file 1 [file plants-14-03736-s001.zip › plants-3982565-supplementary.pdf]

Supplementary Materials:

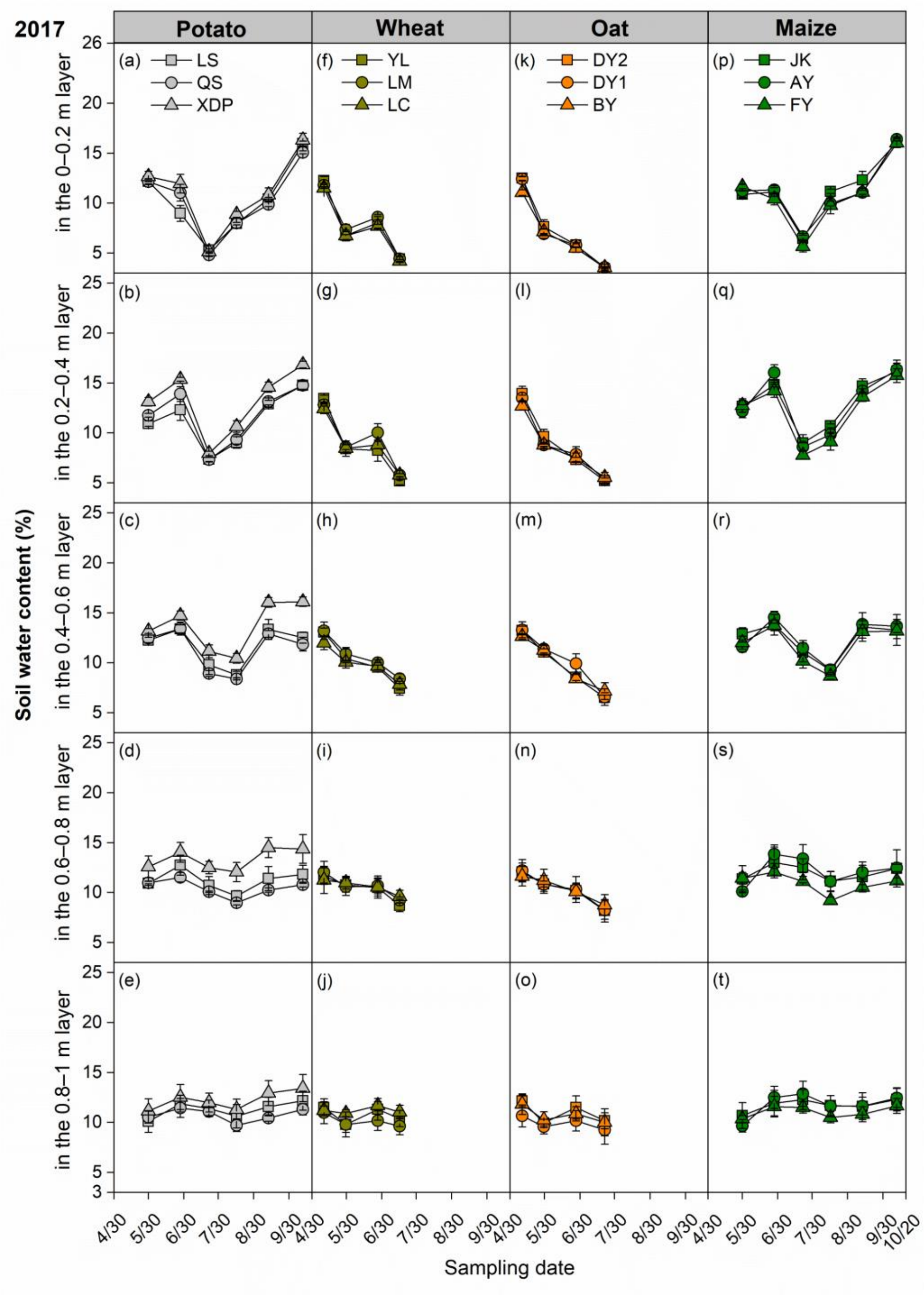

Figure S1. Soil water content dynamics in the 0–0.2 m, 0.2–0.4 m, 0.4–0.6 m, 0.6–0.8 m, 0.8–1 m layers for potato (**a–e**), wheat (**f–j**), oat (**k–o**), and maize (**p–t**) cultivars throughout the 2017 growing season. Bars represent ± one standard error (n = 3).

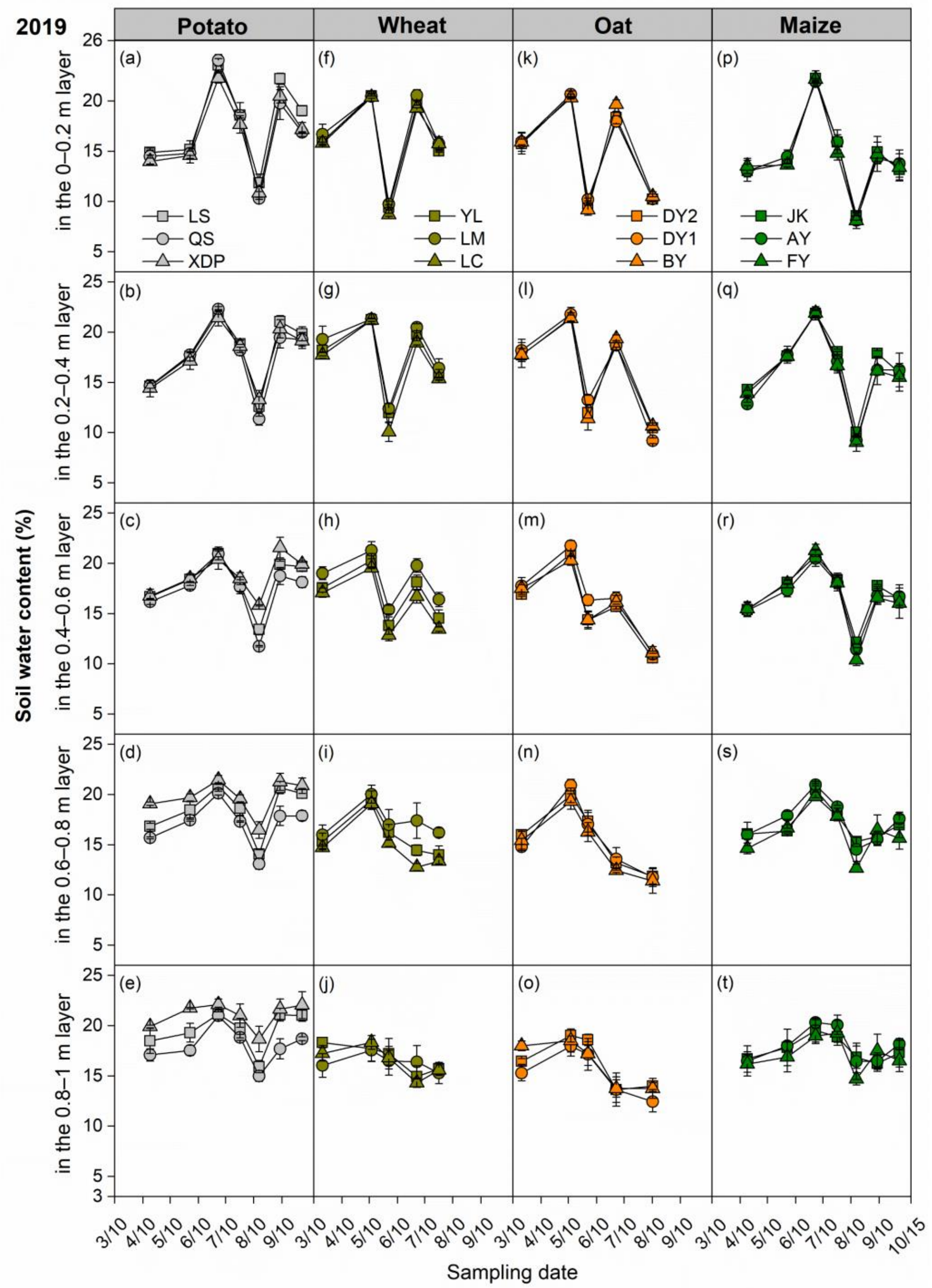

Figure S2. Soil water content dynamics in the 0–0.2 m, 0.2–0.4 m, 0.4–0.6 m, 0.6–0.8 m, 0.8–1 m layers for potato (**a**–**e**), wheat (**f**–**j**), oat (**k**–**o**), and maize (**p**–**t**) cultivars throughout the 2019 growing season. Bars represent ± one standard error (n = 3).

Table S1. Two-way ANOVA analysis of soil water storage (SWS) in the 0–2 m profile at sowing and harvest, change in SWS between sowing and harvest (ΔSWS), total seasonal evapotranspiration (ET), and ET rate under cultivation of potato, wheat, oat, and maize in 2017–2019 cropping years.

Crops	Factors	SWS at sowing	SWS at harvest	ΔSWS	ET	ET rate
Potato	Year (Y)	***	***	***	***	***
	Cultivar (C)	*	**	**	**	**
	Y × C	*	*	***	***	***
Wheat	Year (Y)	***	***	***	***	***
	Cultivar (C)	ns	*	ns	ns	ns
	Y × C	ns	ns	ns	ns	ns
Oat	Year (Y)	***	***	***	***	***
	Cultivar (C)	ns	ns	ns	ns	Ns
	Y × C	ns	ns	ns	ns	ns
Maize	Year (Y)	***	***	***	***	***
	Cultivar (C)	ns	*	***	***	***
	Y × C	ns	ns	ns	ns	ns

*, $P < 0.05$; **, $P < 0.01$; ***, $P < 0.001$; ns, not significant.
